# Involved *microRNAs* in alternative polyadenylation intervene in breast cancer via regulation of cleavage factor “*CFIm25*”

**DOI:** 10.1038/s41598-020-68406-3

**Published:** 2020-07-14

**Authors:** Mona Tamaddon, Gelareh Shokri, Seyed Mohammad Ali Hosseini Rad, Iman Rad, Àmirnader Emami Razavi, Fatemeh Kouhkan

**Affiliations:** 1 Stem Cell Technology Research Center, No. 9, East 2nd, St., Farhang Blvd., Saadat Abad St., Tehran, 1997775555 Iran; 2 Department of Microbiology and Immunology, University of Otago, Dunedin, Otago 9010 New Zealand; 3 Ìran National Tumor Bank, Cancer Biology Research Center, Cancer Institute of Iran, Tehran University of Medical Sciences, Tehran, Iran

**Keywords:** Cancer, Cell biology, Computational biology and bioinformatics, Genetics

## Abstract

Cleavage factor “CFIm25”, as a key repressor at proximal poly (A) site, negatively correlates to cell proliferation and tumorigenicity in various cancers. Hence, understanding CFIm25 mechanism of action in breast cancer would be a great benefit. To this aim four steps were designed. First, potential *miRNAs* that target 3′-UTR of *CFIm25* mRNA, retrieved from Targetscan web server. Second, screened *miRNAs* were profiled in 100 breast cancer and 100 normal adjacent samples. Third, *miRNAs* that their expression was inversely correlated to the CFIm25, overexpressed in MDA-MB-231 cell line, and their effect on proliferation and migration monitored via MTT and wound healing assays, respectively. Fourth, interaction of *miRNAs* of interest with 3′-UTR of *CFIm25* confirmed via luciferase assay and western blot. Our results indicate that *CFIm25* considerably down-regulates in human breast cancer tissue. qRT-PCR assay, luciferase test, and western blotting confirm that *CFIm25* itself could be directly regulated by oncomiRs such as *miR-23*, *-24, -27, -135, -182* and *-374*. Besides, according to MTT and wound healing assays of cell lines, *CFIm25* knockdown intensifies cell growth, proliferation and migration. Our results also confirm indirect impact of CFIm25 on regulation of mRNA’s 3′–UTR length, which then control corresponding *miRNAs’* action. *miRNAs* directly control CFIm25 expression level, which then tunes expression of the oncogenes and tumor proliferation. Therefore, regulation of CFIm25 expression level via *miRNAs* is expected to improve treatment responses in breast cancer.

## Introduction

Living organisms apply complicated mechanisms of gene regulation to generate different cell types, which result in various behaviors from a single genome^1,2^. Dynamic and highly polymorphic nature of mRNA polyadenylation, as a functional aspect of gene regulation, is one of the most recent discoveries in this field^3,4^. Cleavage and polyadenylation of nascent mRNA accomplishes via application of two large multimeric complexes, which named as cleavage–polyadenylation specificity factor (CPSF) and cleavage stimulation factor (CstF), respectively. The CPSF recognizes polyadenylation site (PAS) element located ~ 19 nucleotides away from the polyA site in the 3′-UTR of mRNAs, while CstF directly interacts with CPSF and binds to GU-rich element at downstream of cleavage site^5–7^. Most human genes harbor multiple polyadenylation signals, which results in variable 3′-UTR length without changing the coding region, or produce different protein isoforms through the usage of poly(A) sites residing in internal introns/exons^3,8–10^.

The UTR-alternative polyadenylation^1^ site play critical roles in various biological networks through changes that occur in 3′-UTR length. For example, UTR-APA could influence the stability, translation efficiency and subcellular localization of mRNA. The UTR-APA also regulates protein transport by altering interaction of RNA binding proteins with the 3′-UTR. Therefore, UTR-APA site quantitatively affects corresponding protein expression through a complicated mechanism^10,11^. UTR-APA site controls length of the 3′-UTR, which then controls *miRNAs*’ binding sites availability at the 3′-UTR. The choice of poly (A) site can be influenced by a key factor in APA, which is human cleavage factor Im (CFIm)^12^.

The CFIm is an essential component in the pre-mRNA 3′-UTR processing complex. It contains three polypeptides of 25, 59 and 68 kDa that are designated as CFIm25 (CPSF5/NUDT21), CFIm59 (CPSF7, cleavage and polyadenylation specificity factor 7), and CFIm68 (or CPSF6). Diminution of CFIm25 level induces a global recruitment of weak polyA, placed completely proximal to the stop codon, while enhances cell proliferation. Previous studies confirmed down-regulation of CFIm25 in tumor tissue compared to the normal tissue. Besides, diminution of CFIm25 level comes up with shorter length of 3′-UTR in various oncogenes and induces proliferation of tumor cells^13–15^. However, mechanism that regulates CFIm25 expression, which then correlates to the occurrence and development of cancer, remains unknown.

*miRNAs* are tiny non-coding RNAs that are involved in post-transcriptional gene regulation. They target cognate mRNAs contain complementary sequences in their 3′-UTR, which either prevents mRNA translation or induce its degradation^16–18^. Numerous *miRNAs* have been shown to be aberrantly expressed, and act as oncogenes in several types of cancer such as breast cancer^19–23^. Besides, *miRNAs* mimic tumor suppressor roles in many cases. That’s why loss of *miRNA* expression correlates with inception or the aggressiveness of tumors^24,25^.

In this study, we checked if *miRNAs* are involved with regulation of CFIm25 expression. To examine regulatory impact of *miRNAs* on endogenously APA master regulator CFIm25, correlation of oncogenic *miRNAs* with CFIm25 investigated in clinical samples of breast cancer patients. Then, we explored impact of transfected *miRNAs* in breast cancer cell line, in a set of parallel separate experiments. Identification of numerous oncomiRs that suppress CFIm25, which then induce proximal polyadenylation and mediate in production of shortened oncogenes in breast cancer, confirms involvement of *miRNAs* in regulation of CFIm25.

## Results

### Correlation of CFIm25 expression level with oncogenic *miRNAs* in breast cancer clinical samples and cell lines

**Figure 1:**
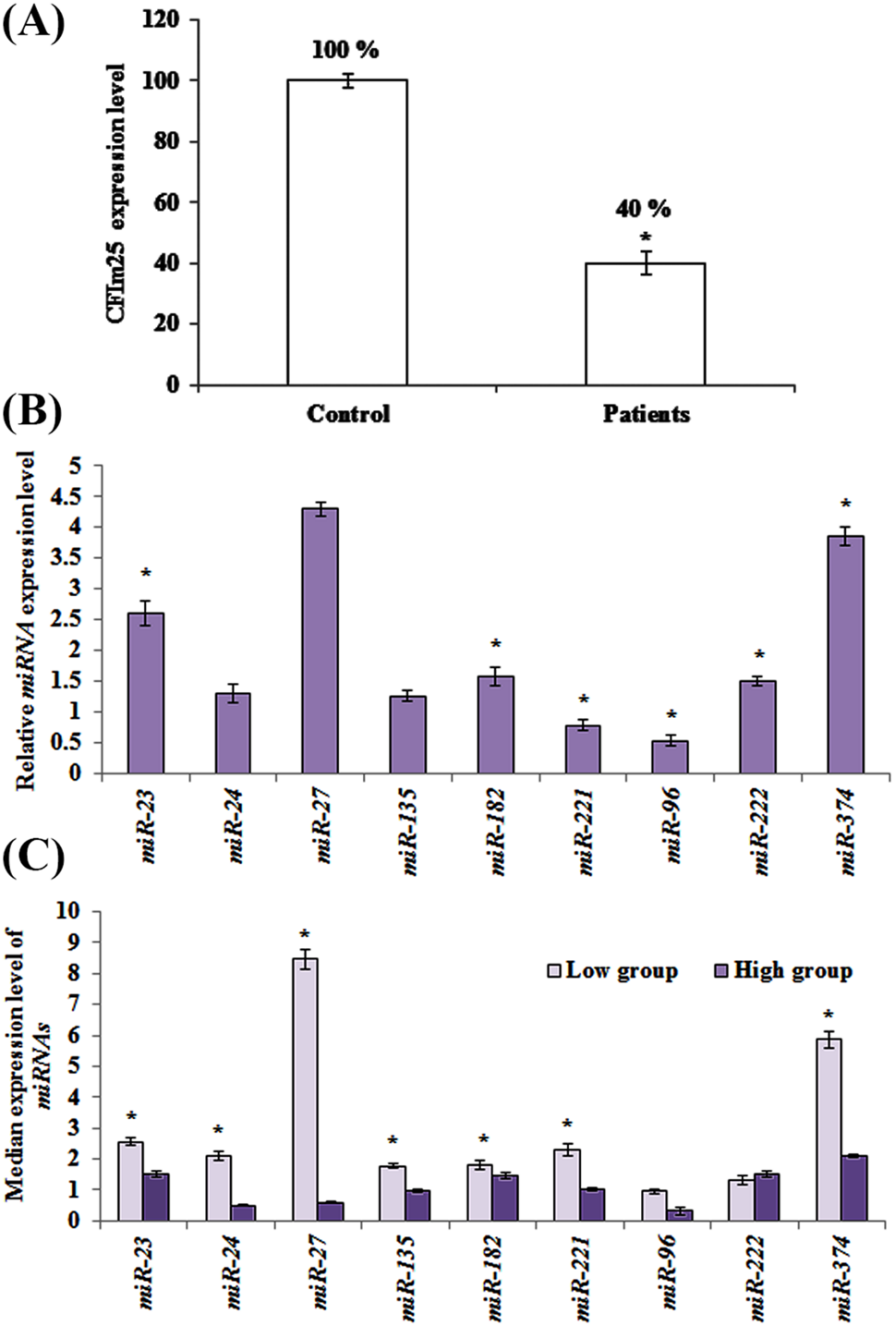
Comparative expression of CFIm25 vs involved *miRNAs*. (**a**) CFIm25 expression level in samples acquired from breast cancer patients and control group. CFIm25 is significantly lower in patients compared to controls. (**b**) Relative gene expression of *miRNAs* in patients. (**c**) Distribution of *miRNA* expression in patients according to low and high expression levels of CFIm25. Patients categorized as low or high CFIm25‑expressing tumors, using median expression of CFIm25 in normal samples as a cut-off. Data represent mean of at least three independent experiments. The star above the bars, represents significant difference respect to the corresponding control reference. The results are relative gene expression after normalization with *β-actin* for *CFIm25* and *SNORD47* for *miRNA* genes, using 2^−ΔΔCt^ method.

According to bioinformatics analysis, *miR-23*, *miR-24*, *miR-27*, *miR-96*, *miR-135*, *miR-182*, *miR-221*, *miR-222* and *miR-374* found to target CFIm25 beside their oncogenic potential. In order to determine whether CFIm25 expression is controlled by *miRNAs* of interest in breast cancer, we measured the expression levels of potentially involved *miRNAs* in breast cancer patients (n = 100) and normal adjacent tissues (n = 100) using *miR*-quantitative qRT–PCR analysis. Results showed considerable elevated level of *miRNAs* in samples acquired from patients (at least in 6 out of 9 monitored *miRNAs*), while CFIm25 expression level was diminished significantly (Fig. 1a, b). In the next step, patients categorized as low or high CFIm25‑expressing tumors, using the median expression for CFIm25 in the normal sample as a cut-off.

Since CFIm25 level differs in samples acquired from patients, their correspondingly correlated *miRNAs* might be variable too. By this regard, distribution of the median expression of nine *miRNAs* of interest evaluated respect to both low and high levels of CFIm25 expression (Fig. 1c).

The median gene expression level of *miR-23*, *miR-24*, *miR-27*, *miR-135*, *miR-182*, *miR-221* and *miR-374* was significantly higher in the patients expressing lower levels of CFIm25, compared to those expressing higher levels (*p* < 0.05).

### Intervention of CFIm25 in length determination of proto-oncogenes’ 3′-UTR

Three out of five proto-oncogenes interact efficiently with CFIm25 and CPSF1 either at proximal or distal of 3′-UTR while the other two proto-oncogenes (*EGFR*: NM_005228.5, *AKT3*: NM_005465.5) interact with both proteins merely at distal of the 3′-UTR (Table 1). Therefore, exclusive interaction of CFIm25/CPSF1 complex with distal region of *EGFR* and *AKT3* 3′-UTR coding mRNA, would guarantee suppression of breast cancer progression.

**Table 1.**
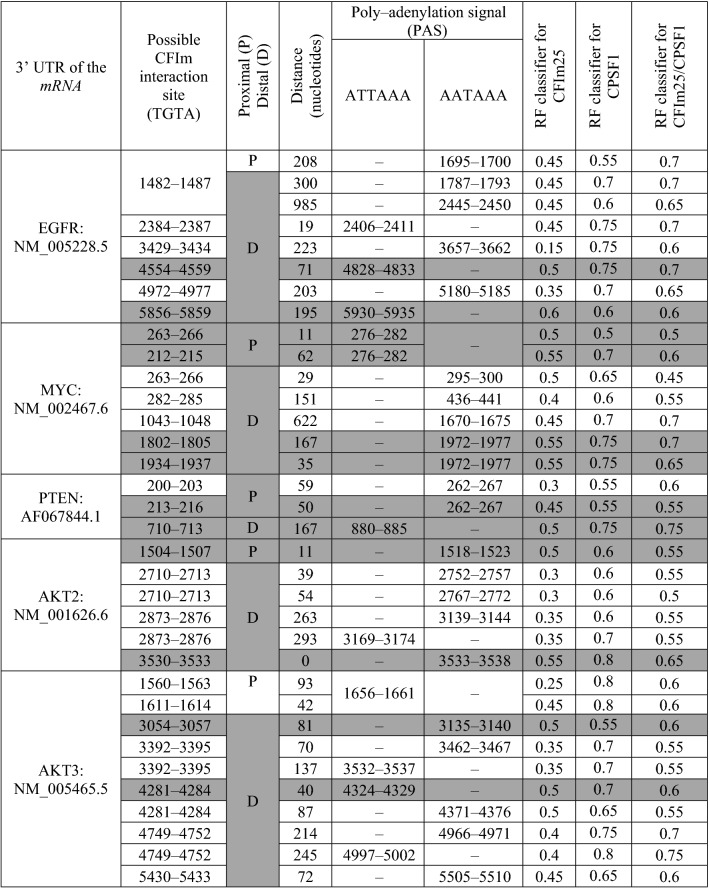
Proto-oncogenes that CFIm25 and CPSF1 target their 3′UTR mRNA.

The cases that are highlighted in dark grey represent proto-oncogenes with positive interaction probability. The RF (Random Forest) classifier is a probability index, which falls between 0 (the lowest probability) to 1 (the highest probability).

### Some of the potentially involved *miRNAs* in breast cancer, down-regulate *CFIm25* in MDA-MB-231 cell line

**Figure 2:**
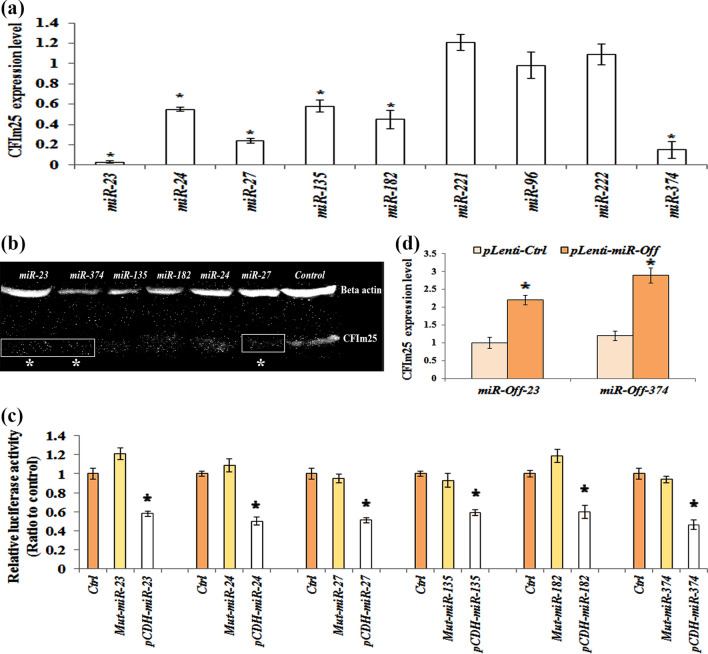
Some of predicted *miRNAs* directly regulate CFIm25 expression. (**a**) CFIm25 expression level in cell line with enforced expressions of *miRNAs* of interest, monitored by Real-time PCR and (**b**) Western blot technique. The western blot bonds cropped from a same gel and membrane. The original image of the membrane and gel could be found in supplementary Figs. 1 and 2. (**c**) Luciferase assay of the HEK293 cells that are transiently transfected with *CFIm25-pSICHECK2* construct and WT-miRNAs/mut-miRNAs. (**d**) Expression level of CFIm25 in cells transducted with either of *miR-Off-23* or *miR-Off-374* constructs. Protein normalization performed against β-actin expression. Columns are mean of three different experiments and error bars are standard deviation (*p* < 0.05). The star represents significant difference respect to the corresponding control reference.

To confirm the impact of *miRNAs* on *CFIm25* regulation in clinical samples, MDA-MB-231 stable cell lines were generated using *miRNA* containing lentivirus transduction. We determined that enforced expressions of *miR-23, miR-24, miR-27, miR-135, miR-182* and *miR-374* significantly down-regulated CFIm25 (*p* < 0.05), while made no change in cells that were transducted with *Ctrl-vector, miR-96, miR-221 and miR-222* (Fig. 2a). Up-regulation of *miR-23* and *miR-374* down-regulated CFIm25 the most, compared to other *miRNAs* including *miR-24, miR-27, miR-135* and *miR-182* (Fig. 2a). According to image analysis of western blot gels via Image J software, CFIm25 protein level notably decreased while *miR-23*, *27*, and *374* were up-regulated (Fig. 2b).

In the next step, we investigated whether 3′-UTR of *CFIm25* has functional targets for predicted *miRNAs*. To this aim, full-length sequence of CFIm25 3′-UTR cloned to psiCHECK-2 vector, which is placed at downstream of Renilla luciferase reporter gene (*CFIm25-psiCHECK2*). In a parallel experiment, as negative controls, the seed sequence of *miR-23*, *miR-24*, *miR-27*, *miR-135*, *miR-182* and *miR-374* mutated intentionally at certain points. The stem-loop structures that formed following introduction of mutations, cloned to *pCDH-TurboGFP* vector (*Mut-miRNAs*) in *shRNA* format. The HEK293 cells transiently transfected with the *CFIm25-pSICHECK2* construct and WT-*miRNAs*, which led to a significant decrease of reporter activity compared to the control (Fig. 2c). Activity of the reporter construct was unaffected by a simultaneous transfection with Mut-*miRNAs* carried a mutated seed sequence. Taken together, these data strongly suggest that *miR-23*, *miR-24*, *miR-27*, *miR-135*, *miR-182* and *miR-374* directly bind to 3′-UTR of *CFIm25*’s mRNA.

According to our findings that suggested *miR-23* and *miR-347* had something to do with CFIm25 down-regulation, we used *miR-Off-23* and *miR-Off-374* to silence *miR-23* and *miR-374*. Now, by using *miR-Off-23* and *miR-Off-374*, we can confirm if silencing of *miR-23* and *miR-374* directly controls overexpression of CFIm25 in breast cancer’s cell line or not. To this aim, *miR-Off-23* and *miR-Off-374* transducted to MDA-MB-231 cell line and efficiency of transduction monitored using qRT–PCR analysis. Transduction of *miR-Off-23* and *miR-Off-374* resulted in significant overexpression of CFIm25 as demonstrated in Fig. 2d (by 3.3 and 2.89 fold increase in case of *miR-Off-23* and *miR-Off -374*, respectively).

### Selected miRNAs can regulate cell proliferation in breast cancer cell line partly by modulating CFIm25 mRNA

**Figure 3:**
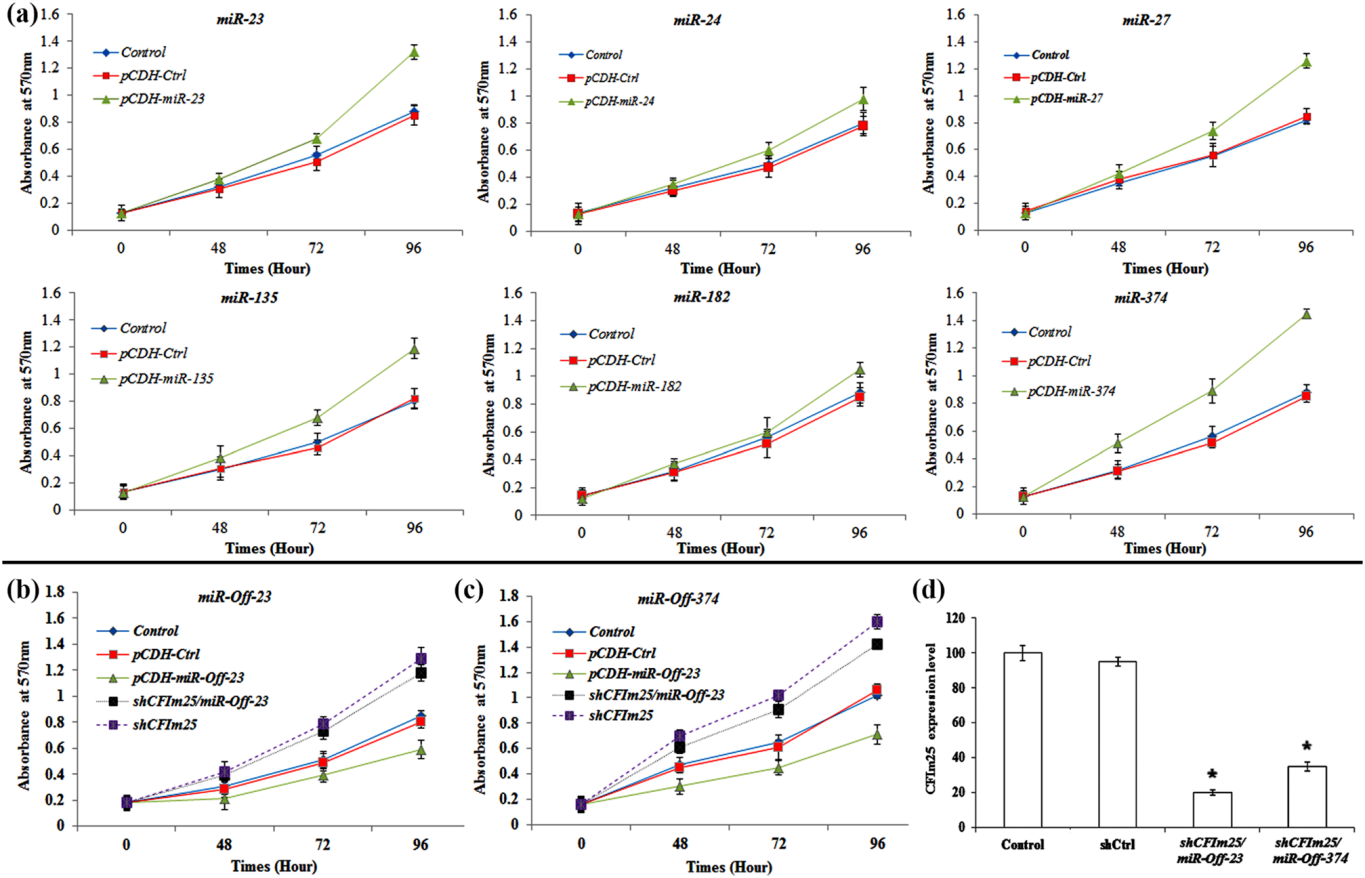
Viability test of MDA-MB-231 cells that were transducted with different cloned *miRNAs*. (**a**) MTT assay of MDA-MB-231 cells that are transducted with *miRNAs* of interest. MTT assay of transducted MDA-MB-231 cells by (**b**) *miR-Off-23* and (**c**) *miR-Off-374* that transfected by *shCFIm25* vector. Each time point was expressed as total absorbance at 570 nm after background subtraction (Y axis). Points are mean of three experiments and error bars represent standard deviation. (**d**) *CFIm25* expression level in *shCFIm25* transfected cells. The star above the bar, represents the significant difference respect to the corresponding control reference.

To determine importance of *CFIm25* in regulation of MDA-MB-231 proliferation, we intentionally performed MTT and wound healing assays in *miRNAs* transduced MDA-MB-231 cells. Result of the MTT assay confirmed increment of “cell proliferation index” in transduced cells as compared with that of the control groups (*p* < 0.05; Fig. 3a).

To clarify direct impact of *miRNAs* on cell proliferation through regulation of 3′-UTR of *CFIm25* mRNA, MDA-MB-231 cells once transduced with *miR-Off-23*/*miR-Off-374* and assessed by MTT assay. In the second approach, transduced cells transfected by *shCFIm25* vector and evaluated by MTT assay. When *miR-Off-23* and *miR-Off-374* transduced to cells and transiently co-transfected with *shCFIm25* vector (Fig. 3b, c), CFIm25 expression level diminished and cell proliferation significantly increased (Fig. 3d). Collectively, these results suggest that *miR-Off-23/-374* vectors up-regulated CFIm25 protein level, which eventually suppressed proliferation of cancer cells.

### Wound healing assay exemplifies CFIm25 balance with *miR-23, -374*

**Figure 4:**
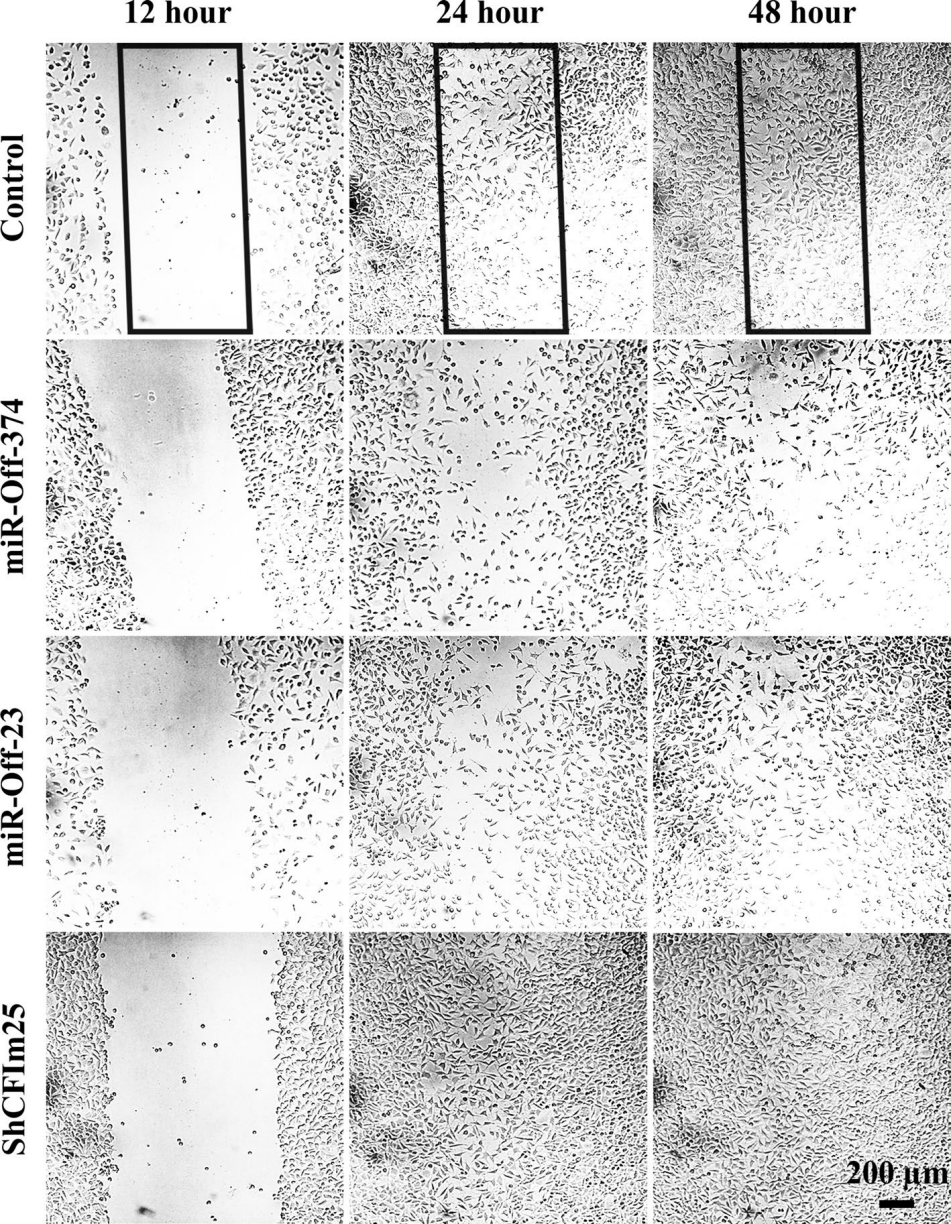
Wound healing assay of transducted MDA-MB-231 cells. Migration of transducted MDA-MB-231 cells toward the scratched area (rectangle) is monitored by 12-h intervals.

The wound healing assay designed to evaluate role of *miR-Off-23*/*miR-Off-374* in MDA-MB-231 in cell migration. As what is shown in Fig. 4, down-regulation of *miR-23* and *miR-374* (by *miR-off-23, -374* transfection) significantly decreased migration ability of transduced cells. However, *shCFIm25* vector improved migration.

**Figure 5:**
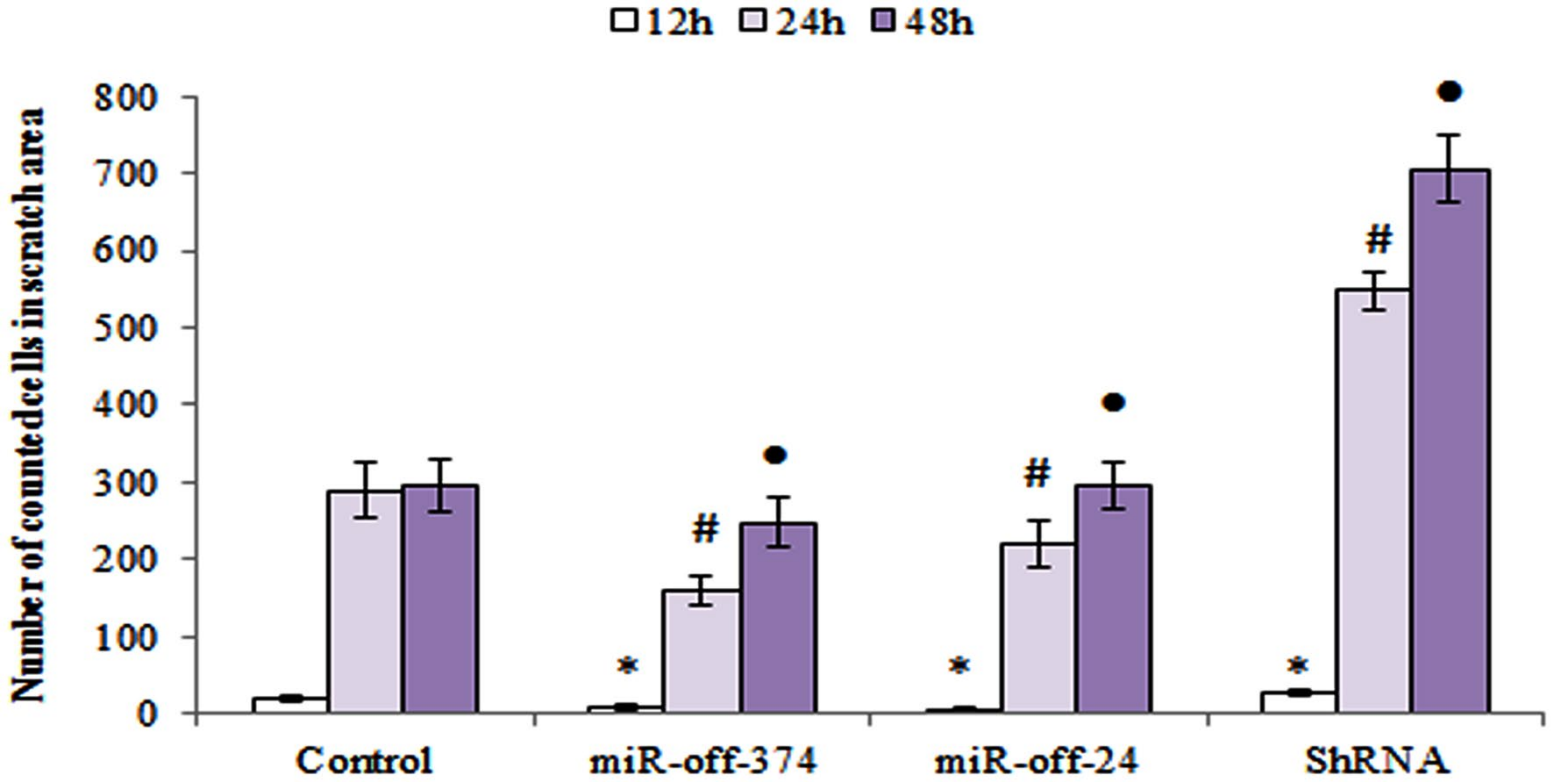
Time course of MDA-MB-231 migration from scratch borders toward the empty area. Diagram of migrated cells to the area of interest that are counted using ImageJ software. Columns are mean of three experiments and error bars represent standard deviation. The (asterisk, hash and filled circle) above the bars, represents the significant difference respect to the control reference at 12, 24 and 48 h, respectively.

Results of migration assay showed that number of migrated cells in the *miR-Off-23*/*miR-Off-374* transduced group, was less than that in the *shCFIm25* group. This last finding confirmed that the migration ability of MDA-MB-231 cells was inhibited by *miR-Off-23*/*miR-Off-374*, which had been found previously to be under influence of *CFIm25* expression level (Fig. 5).

## Discussion

It is conformed that most of the human genes have multiple polyadenylation signals, which dynamically regulate their variations in 3′-UTR length^26^. The *CFIm25*, as a newly discovered repressor of proximal poly (A) site usage, has a significant role in UTR-APA site regulation. The CFIm25, as a subunit of cleavage factor complex, recruits CPSF6 and/or CPSF7 along with CPSF5, and activates 3′-mRNA cleavage site and polyadenylation processing machinery^3,11^. Down-regulation of CFIm25, significantly diminishes “distal poly-A signal” length, and enhances tumorigenic properties and size of the tumor cells. However, CFIm25 overexpression reduces aforementioned properties and inhibits tumor growth. An explanation for this is that by choosing a proximal PAS, the 3′-UTR length of the mRNAs shortens, which then eliminates *miRNAs* binding sites and revokes regulation through *miRNAs*^11,27–29^. Here, we approved *CFIm25* is indirectly involved in regulation of many genes through changing 3′-UTR length, which then eliminates binding sites of their corresponding *miRNAs*. Nonetheless, more studies are still needed to clarify comprehensive regulatory mechanism of *CFIm25* gene by *miRNAs*.

Although breast cancer is the most common cancer diagnosed in women, survival rates of breast cancer have improved recently, which is mostly due to factors such as a new personalized approach to cancer treatment and a better understanding of the molecular mechanism of the disease^30^. In this study, we used human breast cancer specimens to analyze their *CFIm25* expression profile. We confirmed that *CFIm25* is significantly down-regulated in breast cancer samples, compared to the controls. This phenomenon is consistent with the previous study^15^ that introduced CFIm25 as APA regulator that was also down-regulated in glioblastoma and came up with cancer proliferation and tumorigenicity^16^. Besides, it was reported that expression level of *CFIm25* in hepatocellular carcinoma (HCC) was negatively correlated to the metastatic potential of HCC cell line through increasing E-cadherin level^31^, while glutaminase *mRNA* isoforms, which contain distinct 3′-UTR (KGA and GAC), showed a complex interplay between RNA processing and *microRNA* repression in controlling glutamine metabolism in cancer cells^14^.

In the next step of the current study, we selected nine oncomiRs using bioinformatics analysis. They all confirmed to have recognition sites in the 3′-UTR of the *CFIm25* (*miR-23*: 2 sites, *miR-24*: 2 sites, *miR-27*: 3 sites, *miR-96*: 1 site, *miR-135*: 2 sites, *miR-182*: 1 site, *miR-221*: 2 sites, *miR-222*: 2 sites and *miR-374*: 4 sites). So far, several studies investigated role of oncomiRs in different cancers including breast cancer^20,24^. However, no experimental work has performed to clarify effects of these *miRNAs* on CFIm25 expression level and, as a result, in regulation of UTR-APA site. When clinical samples divided into two groups, whose CFIm25 expression was more or lower than the median expression of control samples, a negative correlation between level of *miR-23*, *miR-24*, *miR-27*, *miR-135*, *miR-182* and *miR-374* and CFIm25’s expression was found. This negative correlation in turn suggests that *CFIm25* is being regulated by aforementioned *miRNAs*. Besides, in vitro data from transduced MDA-MB-231 cells confirmed that high levels of *miR-23, miR-24, miR-27, miR-135, miR-182* and *miR-374* was negatively correlated with either *CFIm25* mRNA or its protein content.

According to previous reports, low level of CFIm25 leads to cancer proliferation and migration^14,28,31^, which is in agreement with our MTT and wound healing assay results. Importantly, functional regulatory role of *miR-23* and *miR-374* on *CFIm25* mRNA, approved via MTT assay. This assay confirmed increased growth of MDA-MB-231 cells, which were co-transduced with *miR-Off-23*/*miR-Off-374* and *CFIm25* knock-down vectors. CFIm25 is known to be crucial in controlling invasion and metastasis of HCC^32^. It is also confirmed that activation of APA sites can affect the migratory capacity of cancer cells^33^ as well as fibroblasts^34^. According to the results of wound healing assay, regulation of CFIm25 expression by miR-23 and 374 directly controls breast cancer cells’ migration and invasion. Since invasion and metastasis are the underlying causes of poor long-term survival of breast cancer patients^35^, regulation of CFIm25 via miRs could be considered as a promising strategy for inhibition of metastasis in future clinical studies.

Parental genes of mRNAs with shorter 3′-UTRs, are more prone to up-regulation during tumorigenesis due to increased escaping from *miRNA* repression. It is determined that almost 67% genes with shorter 3′-UTRs in tumors, have lost at least one predicted miRNA-binding site^36^. In some cases, overexpression of oncogenic proteins comes up without regulatory intervenience of the proto-oncogenes. One example of this phenomenon is cancer cell lines (such as MDA-MB-231) that express substantial amounts of mRNA isoforms with shortened 3′-UTRs^30,31^. CFIm25 is a regulatory protein that controls key factors such as *EGFR* and *MYC* that are involved in NF-κB and MAPK/ERK pathways^31,37,38^. Our finding suggests that regulation of proto-oncogenes by CFIm25, could be mediated via APA and shortening of 3′UTR of their corresponding mRNAs, which eventually result in progression of breast cancer. The *MYC*, *PTEN* and *AKT2* proto-oncogenes are more responsive to CFIm25 than *EGFR* and *AKT3*, since they have several potential interaction sites either at proximal or distal of their mRNA’s 3′-UTRs (Table 1). Tuning impact of CFIm25 on *MYC*, *PTEN* and *AKT2* will then control downstream phenomena such as glutamine metabolism in cancer cells^14^, which could be applied as an strategy for targeted therapy^39^. Besides, 3′-UTR of *EGFR*’s mRNA contain multiple *microRNA* target sites, which are associated with cell cycle arrest and cell death. As long as CFIm25 interacts with distal region of *EGFR*’s 3′-UTR mRNA (Table 1), suppression of breast cancer tumorigenicity by *miRNAs* is highly plausible^40^. In a similar way, interaction of CFIm25 with distal region of *AKT3* 3′-UTR mRNA, facilitates down-regulation of *AKT3*, which then regulates migration and metastasis in breast cancer cells^41^. These two examples can clarify how CFIm25 interaction with distal region of *EGFR* and *AKT3* 3′-UTR mRNAs, can facilitates suppression of breast cancer tumorigenicity and metastasis, respectively.

## Conclusion

In summary, CFIm25 is down-regulated in human breast cancer tissues compared to the adjacent non-cancerous breast tissues. Besides, *CFIm25* mRNA is also regulated by several *miRNAs* including *miR-23, miR-24, miR-27, miR-135, miR-182* and *miR-374*. Since CFIm25 is a key factor in cancer proliferation, identification of its modulators, such as *miRNAs*, facilitates development of molecular-targeted therapeutics for various cancers including breast cancer. Our in-vitro results confirm functionality of certain *miRNA*s toward regulation of 3′-UTR via CFIm25 in breast cancer cells, which then introduce it as an emerging field of study in pre-clinical studies of breast cancer.

## Methods

### Cell lines and patient sample collection

Hundred fresh frozen samples of human breast cancer tumors and their normal adjacent tissue obtained from the Iran national tumor bank of Cancer Institute (Imam Khomeini hospital, Tehran University of medical sciences, Tehran, Iran). Sample collection performed according to the international tumor bank SOP protocol and written informed consent obtained from patients. This study was approved by the cancer institute of Imam Khomeini Hospital, Tehran, Iran. SOP protocol and consent designed in accordance with ethical standards of Tehran University of medical sciences and with the 1964 Helsinki declaration and its later amendments or comparable ethical standards.

Human embryonic kidney cell line HEK293T (RRID:CVCL_0063) and human breast cancer cell line MDA-MB-231 (RRID:CVCL_0062) were obtained from Pasteur Institute of Iran (IPI) and Iranian biological resource center, respectively. Aforementioned institutes check the authenticity of human cell lines via DNA profiling annually. The obtained cell lines cultured in Dulbecco’s modified Eagle’s Medium (DMEM; Gibco), supplemented with 10% fetal bovine serum (FBS; Gibco) and 1% penicillin–streptomycin (Gibco) at 37 °C with 5% CO_2_ incubation.

### Bioinformatic analysis

The *miRNAs* that were susceptible to target *CFIm25* mRNA 3′-UTR acquired using prediction web servers such as TargetScan, miRWalk (version 3.0) and DianaTool. The standard cutoff for screening predicted *miRNAs* was 8 mer, at the most, while the percentage of context ++ score (CS) did not exceed 95%. Predicted *miRNAs* with the high score were investigated in the miRCancer database. Finally, miRNAs with the oncogenic roles were selected for the rest of the research, including *miR-23, miR-24, miR-27, miR-96, miR-135, miR-182, miR-221, miR-222* and *miR-374*.

In order to find out if CFIm25 efficiently targets 3′-UTR of proto-oncogenes’ mRNAs, three steps were followed. First, 3′-UTR mRNA of twenty proto-oncogenes that known to be involved in breast cancer^42^, retrieved from UTR database^43^. Second, twenty retrieved proto-oncogenes including; NM_053056.2, NM_001759.4, NM_000077.4, JX391994.1, S67388.1, NM_005163.2, NM_001626.6, NM_005465.5, BC040540.1, AF067844.1, L78833.1, U43746.1, KX710182.1, XM_011512894.2, XM_024450643.1, NM_002467.6, NM_002745.4, NM_005343.4, NM_005228.5 and NM_005417.4, were tested as a matter of proximal or distal placement of CPSF interaction site at polyadenylation signal (A[A/T]TAAA) and their neighbor CFIm25 interaction site (TGTA). The PAS that is closest to the 5′ end of the 3′-UTR is considered as “proximal” and the other PAS(s) in the downstream of 3′-UTR considered as distal^44^. Third, the retrieved 3′-UTRs beside CFIm25 (O43809) and CPSF1 (Q10570) submitted to the RNA–Protein Interaction Prediction (RPISeq) server to estimate their interaction probabilities^45^. Interaction probabilities ranged from zero to one. Optimal RNA/protein interactions were the ones with interaction probabilities of more than 0.5, which represented accuracy of the Random Forest (RF) classifiers in cross-validation evaluation experiments on benchmark datasets.

### RNA extraction and qRT-PCR

Total RNA extraction of cell line, human breast cancer tissues and normal specimens was performed using Trizol (Invitrogen) in accordance with manufacturer’s protocol and previous study^22^. Then, 500 ng of total RNA was reverse transcribed to synthesize cDNA with random hexamer primers and Fermentas kit (for *CFIm25* gene) or BON RT adaptor primer and BONmiR kit (for *miRNA* genes). A total of 1 μL of cDNA was amplified using SYBR Green Real-time PCR Master Mix (Takara) and performed on ABI 157 PRISM 7,500 real-time PCR System (Applied Biosystems). The relative expression levels of the CFIm25 and *miRNA* genes calculated using the 2^−∆∆Ct^ method, with normalization against *β-actin* and *SNORD47* internal controls, respectively. Three biological replicates were performed for each set of experiments. The qRT-PCR primer sequences are listed in supplementary Table 1 and 2.

In clinical samples, the mean of CFIm25 and β-actin Cts between all of the normal samples was determined and compared with the obtained Cts of each patient. This procedure adopted from our previous studies with minor modifications^20,46^.

Based on the results, tumors were classified as low and high/equal *CFIm25*‑expressing tumors using the median of the normal samples as a cut-off. Next, the relative gene expression of predicted *miRNAs* in two groups of patients was calculated by comparing obtained Cts with the mean Cts of normal samples.

### Plasmids, viral vectors construction and luciferase assay

For the construction of *miRNA*-expressing vectors, genomic fragment consists of the stem-loop structure of each *miRNA* and theirs flanking genomic sequences, cloned into the mammalian expression vector *pLEX.jred* and *pCDH.TurboGFP* at downstream of the cytomegalovirus (CMV) promoter. The PCR primers used for *miRNAs* cloning were listed in supplementary Table 2. Empty vector without any cloned sequence (*pLEX-Ctrl*/ *pCDH-Ctrl*) was used as control in accordance with *Furukawa *et al. method^47^.

HEK293T cells were transiently co-transfected by *miRNA*-expressing lentivectors (or empty backbones), *pPAX2* plasmid (packaging plasmid), and *pMDG* plasmid (containing *vsv-G*) using calcium phosphate. Lentivirus supernatants were harvested for two or three times, every 12 h, concentrated by ultracentrifuge at 47,000×*g* for 2 h at 4 °C. Lentivirus titer examined by flow cytometry analysis of reporter positive 293 T cells (Attune Acoustic Focusing Flow Cytometer, ABI, FlowJo software)^48^.

To down-regulate *miR-23* and *miR-374* expression, *pLenti-III-miR-Off* constructs containing GFP marker purchased from ABM Company and packaged into lentiviruses particles. For luciferase assay, full length of *CFIm25* 3′-UTR amplified by PCR using the forward 5′-GCAAGCTTAGCTGTTCTTCTGCC-3′ and reverse 5′-GCACTAGTGTCATCAAAATATTTATTAA-3′, respectively. The *CFIm25* 3′-UTR was then cloned to downstream of the luciferase gene in the *pSICHECK2* vector (Promega). Mutant stem-loop forms of each *miRNA*, harboring two mutations in seed sequence, cloned in the lentiviral vector and named Mut-miR, which was considered as negative control. The HEK293 cells were transiently transfected with wild type or mutant *miRNA*-expressing vector and *CFIm25-pSICHECK2* vector using lipofectamine 2000 (Invitrogen)^49^.

Luciferase assay performed after 48 h of post-transfection, using the dual-luciferase reporter assay system (Promega). Renilla luciferase signal normalized against firefly luciferase activity to monitor transfection efficiency. Data are the means of experiments performed in triplicate.

### Western blot

The MDA-MB-231 cells were lysed using cell lysis buffer (50 Mm Tris, pH = 8.0, 150 mM NaCl,1% NP-40, 0.5% sodium deoxycholat, 1 mM sodium fluoride, 1 mM sodium orthovanadate, 1 mM EDTA). The polypeptide component were resolved by electrophoresis at 200 V using 12% SDS–polyacrylamide gel electrophoresis (PAGE) transferred into poly vinyl den fluoride membrane and immersed in 5% non-fat milk powder over one hour at room temperature. Upon completion of the transfer, membranes were incubated with CFIm25 and β-actin primary antibodies (Abcam). After washing, membranes probed with horseradish peroxidase-conjugated secondary antibodies (1:1,000, Abcam) and developed for detection by chemiluminescence on Kodak X-film. In order to quantify bands in western blot gel, acquired images of CFIm25 and beta actin bands with quality of 75 out of 100 compared as a matter of expression level, using Image J software. Higher number of averaged white pixels considered as higher amount of expression.

### Short hairpin RNA (*shRNA*) design and experiments

For knockdown endogenous CFIm25, target sequence cloned as *shRNA* format in the *pCDH-turboGFP*. The *shCFIm25* target sequence is 5′-GCCTCATTCTTATTTCAAGAT-3′. An empty *shRNA* vector (*shCtrl*) used as a negative control. The MDA-MB-231cells seeded into a six-well plate and transfected by *pCDH-shCFIm25* or *pCDH-shCtrl* and lipofectamine 3,000 (Invitrogen) according to the manufacturer instruction.

### Cell viability assay

For investigation of cell proliferation, experiments performed into three groups. In the first group, MDA-MB-231 cells transduced with *miR-Off-23*/*miR-Off-374* expressing vectors. In the second group, MDA-MB-231 cells that were co-transduced with *miR-Off-23*/*miR-Off-374* expressing vectors were transfected by *pCDH-shCFIm25*. In the third group, MDA-MB-231 cells transfected merely by *pCDH-shCFIm25*. At 48, 72 and 96 h after transduction/transfection, 10 μL of MTT reagent added into wells and incubated for next three hours. Next, 100 μL DMSO was added and the resulting absorbance measured at 550 nm using a multi-well spectrophotometer (Bio-Tek).

### Wound healing assay

Wound healing assay performed to evaluate MDA-MB-231 cell migration. The MDA-MB-231 cells seeded in 24-well tissue culture plate until their growth rate reached a confluence of ~ 80%. The monolayer scratched gently with a 20-μL pipette tip across the center of the well. After scratching, wells washed twice with medium to remove the detached cells. Cell migration to the scratch area monitored for additional 48 h and photographed every 12 h. Number of cells moved from the border of scratch toward the empty area considered as the “migration index”. Related calculations performed by ImageJ software.

## Supplementary information

Supplementary Information.

## Abbreviations

CPSF: Cleavage and polyadenylation specificity factor
CstF: Cleavage stimulation factor
PAS: Polyadenylation site
3′-UTR: 3′-Untranslated region
UTR-APA: Untranslated region-alternative polyadenylation
*miRNA*/*miR*: MicroRNA
CFIm25: Cleavage factor Im 25
RF: Random forest classifiers
*shRNA*: Short hairpin RNA
HCC: Hepatocellular carcinoma
