# Supplementary material for: Involved microRNAs in alternative polyadenylation intervene in breast cancer via regulation of cleavage factor “CFIm25”
Source: Sci Rep. 2020 Jul 14;10:11608. doi: 10.1038/s41598-020-68406-3 (PMC7360588; doi:10.1038/s41598-020-68406-3)

Involved *MicroRNAs* in alternative polyadenylation intervene in breast cancer via regulation of cleavage factor “*CFIm25*”

Mona Tamaddon^1^, Gelareh Shokri^1^, Seyed mohammad ali Hosseini rad^2^, Iman Rad^1^, Àmirnader Emami razavi^3^ and Fatemeh Kouhkan^1*^

1 - Stem Cell Technology Research Center, Tehran, Iran.

2 - Department of Microbiology and Immunology, University of Otago, Dunedin 9010, Otago, New Zealand.

3 - Ìran National Tumor Bank, Cancer Biology Research Center, Cancer Institute of Iran, Tehran University of Medical Sciences, Tehran, Iran.

***Corresponding author:**

Stem Cell Technology Research Center, Tehran, Iran.

Phone: +98–21–22082120. Fax: +98–21–22341460.

Address: No. 9, East 2 nd, St., Farhang Blvd., Saadat Abad St., Tehran, Iran , Postal code: 1997775555.

ORCID: https://orcid.org/0000-0001-7262-0207

Email: f.kouhkan@yahoo.com, f.kouhkan@stemcellstech.com

Running title: A cleavage factor mechanism of action in breast cancer

­

Supplementary Table 1. Primers for qRT-PCR of genes and miRNAs

| **Genes** | **Primer sequences** |
| --- | --- |
| **hsa-miR-23a** | FW, 5′-ATCACATTGCCAGGGATTT-3 |
| **hsa-miR-27a** | FW, 5′-CCG TTC ACA GTG GCT AAG -3′; |
| **hsa-miR- 24** | FW, 5′-ACA TGG CTC AGT TCA GCA -3′ |
| **Hsa-mir-135a** | FW, 5′-CGA TAT GGC TTT TTA TTC CTA ′; |
| **Hsa-miR-** **182** | FW, 5 -GGT TTG GCA ATG GTA GAA C -3′; |
| **Hsa-miR-374-5p** | FW, 5′-ACGCTGGATAATACAACCTG -3′; |
| **SNORD47** | FW, 5′-ATC ACT GTA AAA CCG TTC CA -3′; |
| **CFIm25** | FW, 5′-AAC GCT TAA TGA CAG AGA TAC -3′;  RW, 5′-TAT ATG GAT ACT GAG GAG GTT C -3′ |

Supplementary Table 2. Primers for miRNA cloning

| **Genes** | **Primer sequences** |
| --- | --- |
| **hsa-miR-24** | FW, 5′-CGT CTA GAT GTG GTA GCT CAT GGC TGT G -3′;  RW, 5′-TCG AAT TCG GAG CAC ATG CAG ATG ACT G -3′ |
| **hsa-miR-27a** | FW, 5′-CGT CTA GAT TCC AAC CGA CCC TGA GC -3′;  RW, 5′-TCG AAT TCC CTA TCT ATG CTG GCA CCA CA -3′ |
| **hsa-miR-135** | FW, 5′-CGT CTA GAG AAC TGT CTG CAA GCT AGG CT -3′;  RW, 5′-ATG AAT TCG TCC AGA CCC TGA AGT CAG C -3′ |
| **hsa-miR-182** | FW, 5′-AAG CGG CCG CAG GAA GGA CCT TGT CGC AGT-3′;  RW, 5′-CCA CGC GTC ACC TGC CCT CTG CCA CT-3′ |
| **hsa-miR-96** | FW, 5′-GAT CTA GAG AAA CAG GCT GCT TCC AAG-3′;  RW, 5′-CAG AAT TCC CCT GAC ACA AGG ATG CAG-3′ |
| **has- miR-374a** | FW, 5′-AGC TCG AGT GGA AGT CTG TGC ATG GAA G-3′;  RW, 5′-TAT ACG CGT CTG ACA ATC CAG AGC CTG TG-3′ |

Images of western blot bonds were captured by Canon EOS 60D camera in reverse grayscale mode with same setup. Each row (Beta actin, CFIm25) of the bonds belong to separate gel.

The Beta actin western blot bonds were captured in a 2 ˟ 18.1 cm image, while CFIm25 bonds were originally captured in a 2.5 ˟ 18.2 cm image. Captured bond were aligned horizontally without changing the image size. During image processing of figure 2b, resoultion of the picture was set to 400 DPI, bond descriptions were added, and the image resized uniformly using Adobe Photoshop CS6 software. Contrast and brightness were increased and diminished, respectively, by 10 level.

Image processing (changing brightness and contrast) is applied equally across the entire original image and applied equally to the controls. The following image (Supplementary figure 1) represents the ladder of the western blot gel, which is taken using visible light.

Supplementary figure 1


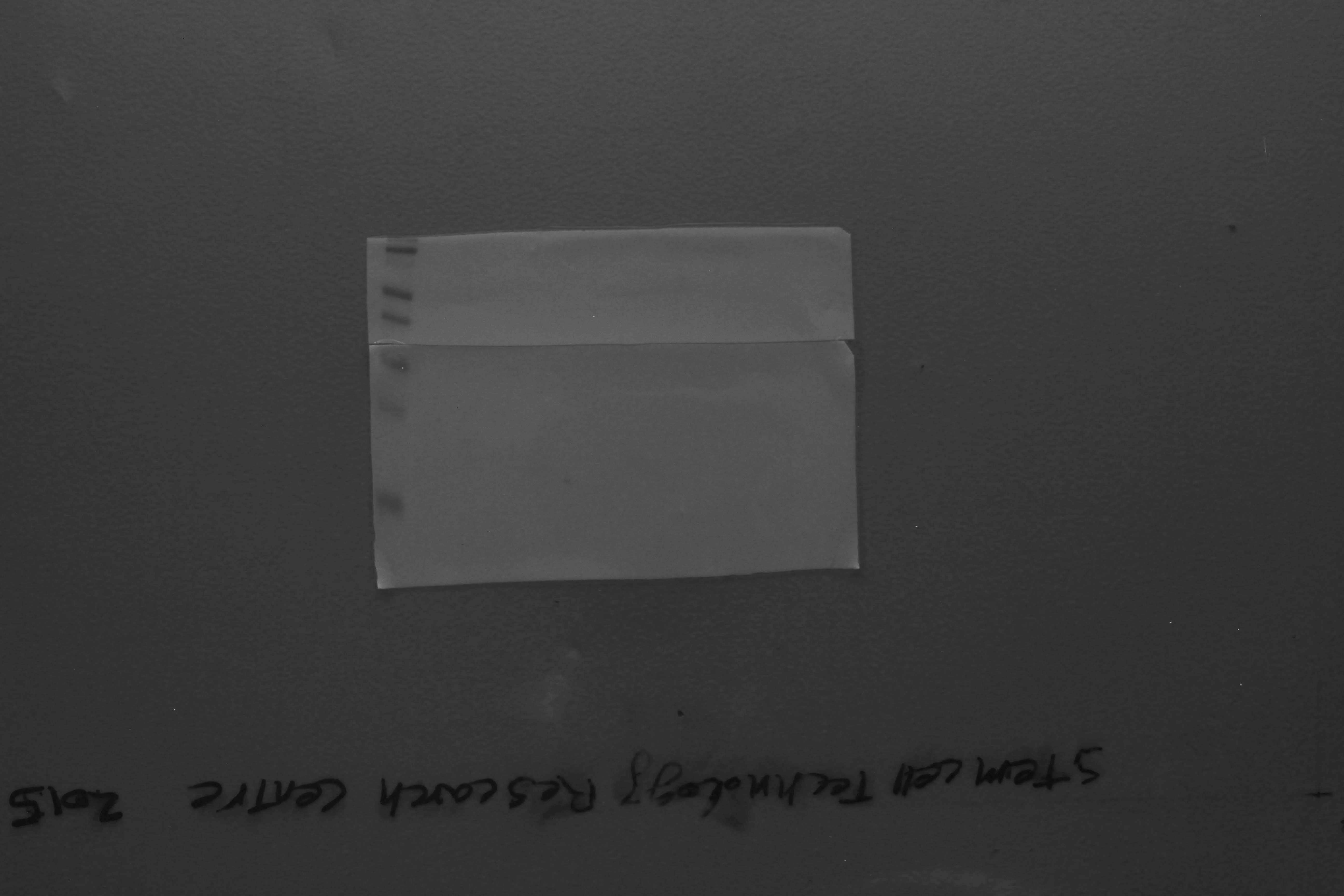


Contrast was adjusted in a way that no data disappeared. Biased choice of threshold settings was not applied to the final images. However, the contrast and brightness were set three times to eliminate white dots. Distance between beta actin and CFIm25 is what has been in the original image. The following image (Supplementary figure 2) represents the original western blot that is taken following uv light exposure. The ladder was cropped and set along the samples to make sure what was the molecular weight of the bands.

Supplementary figure 2


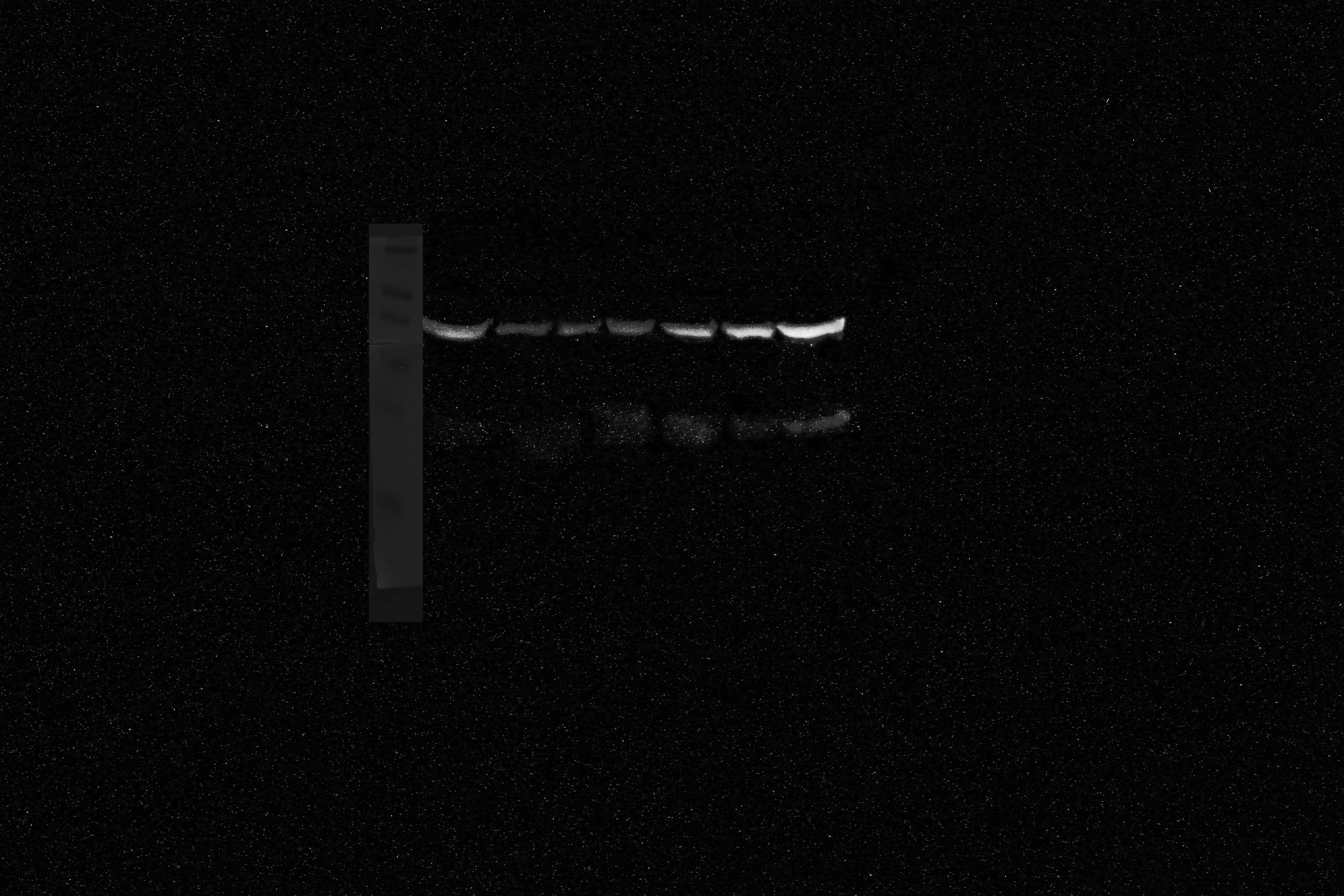


Positive and negative controls, as well as molecular size markers, are included in the same gel and blot. The display of cropped gels and blots is improved in the final version to clarify conciseness of the presentation. It is now mentioned in the figure caption 2 that: “The western blot bonds cropped from a same gel and membrane. The original image of the membrane and gel could be found in supplementary figure 1 and 2. The cropping is mentioned in the figure legend as well as the supplementary information.

Raw material pf wound healing assay

supplementary material

Control: 12 hours after scratch


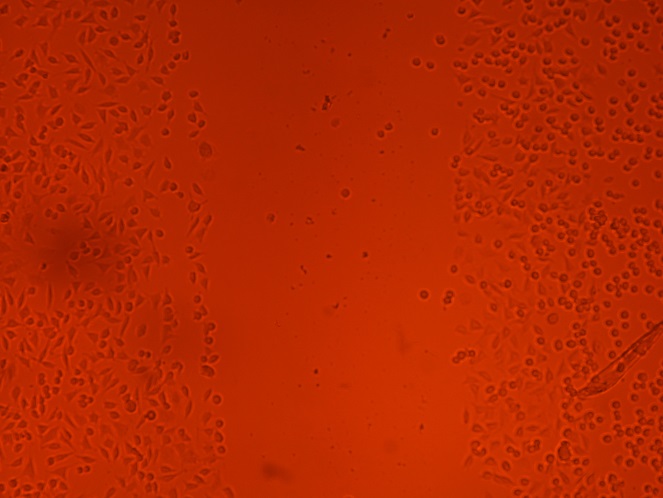


Control: 24 hours after scratch


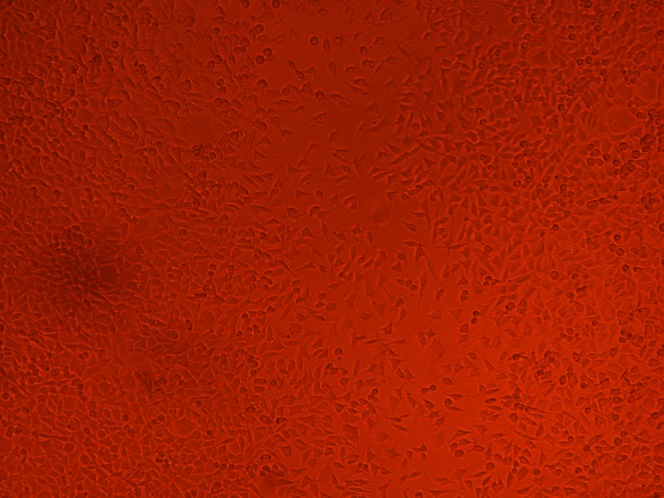


Control: 48 hours after scratch


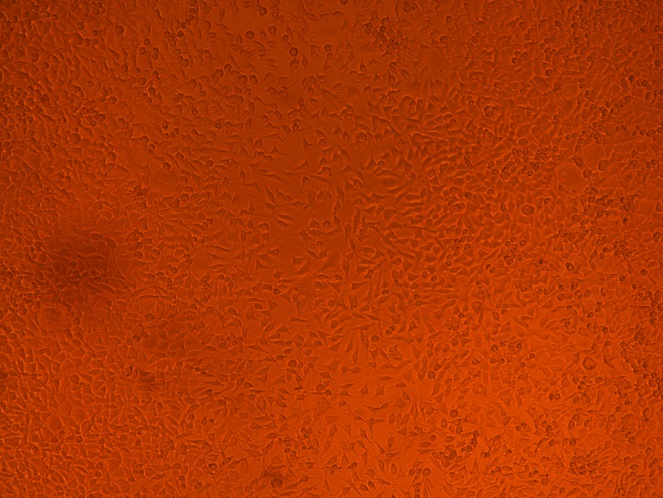


*miR-off-374*: 12 hours after scratch


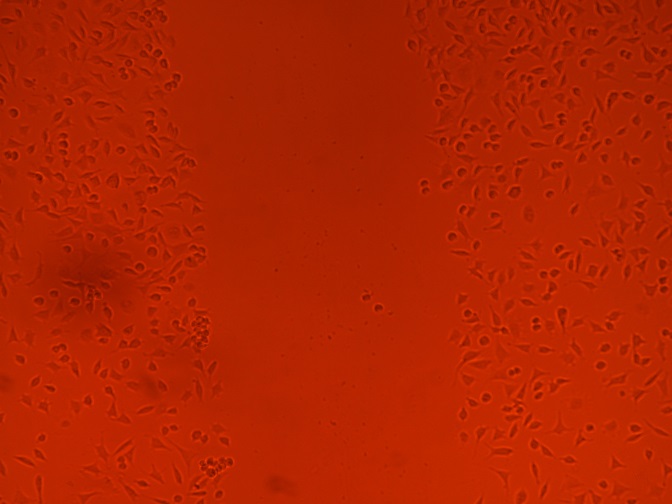


*miR-off-374*: 24 hours after scratch


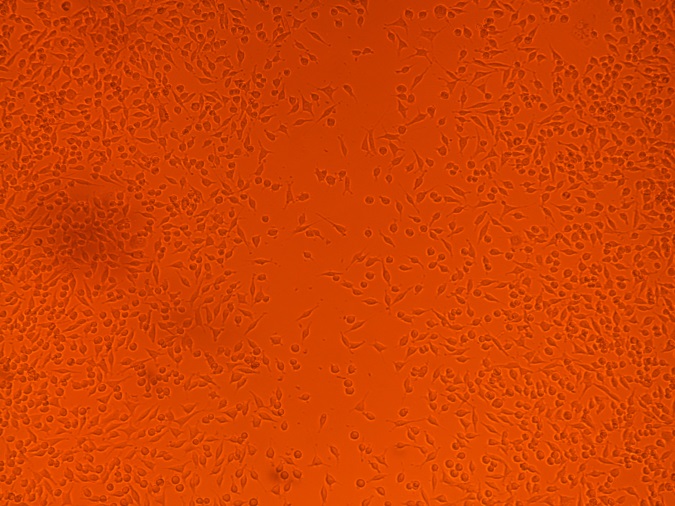


*miR-off-374*: 48 hours after scratch


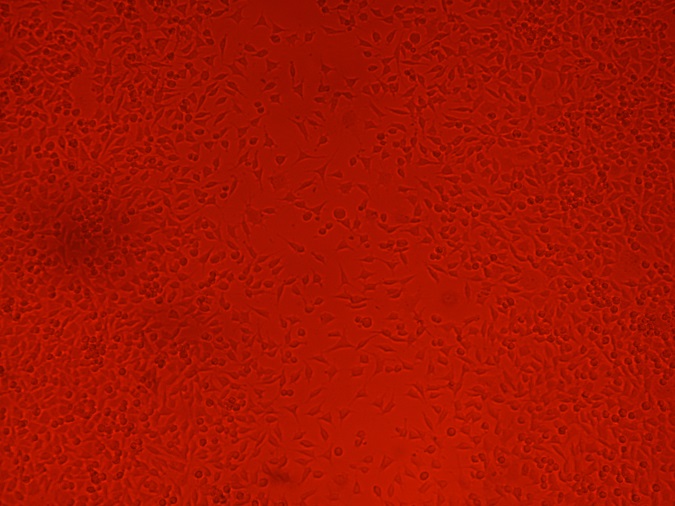


*miR-off-23*: 12 hours after scratch


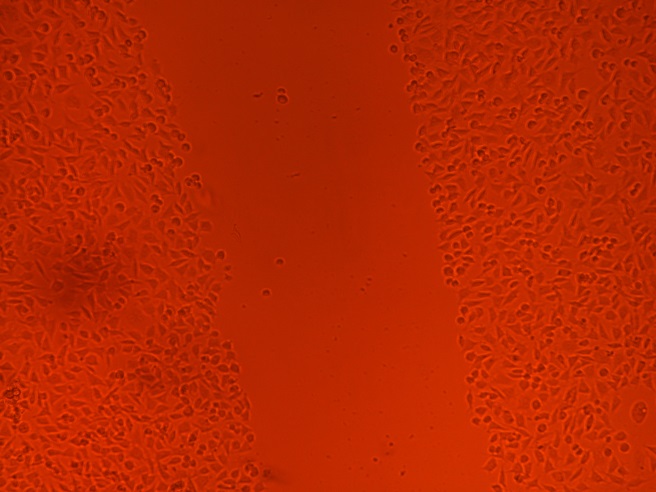


*miR-off-23*: 24 hours after scratch


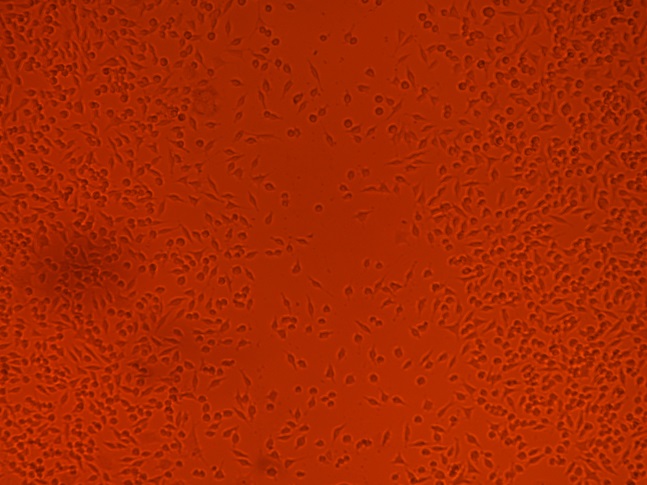


*miR-off-23*: 48 hours after scratch


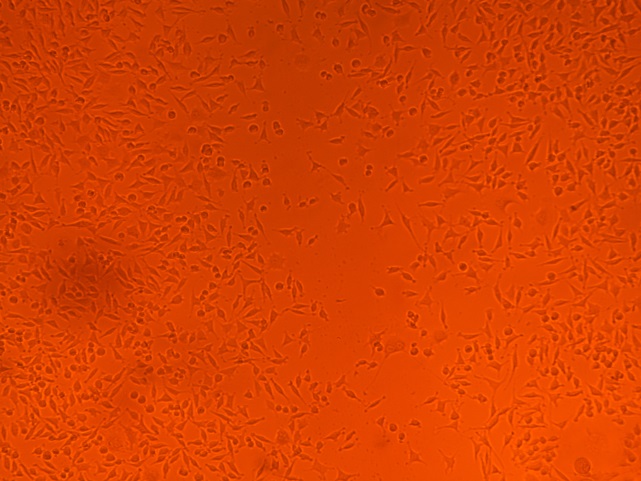


ShCFIm25: 12 hours after scratch


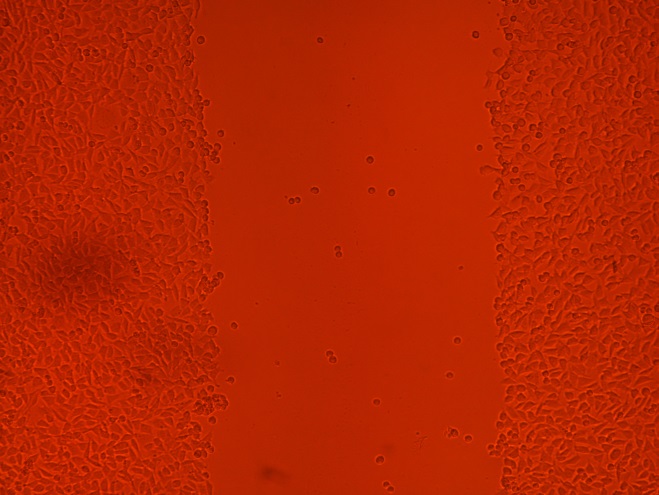


ShCFIm25: 24 hours after scratch


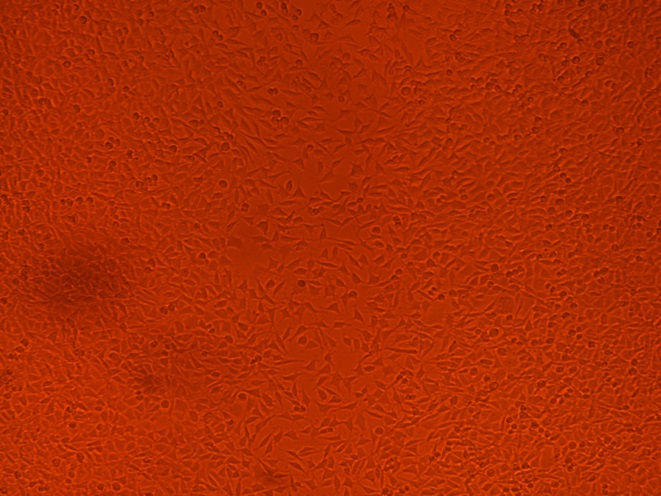


ShCFIm25: 48 hours after scratch


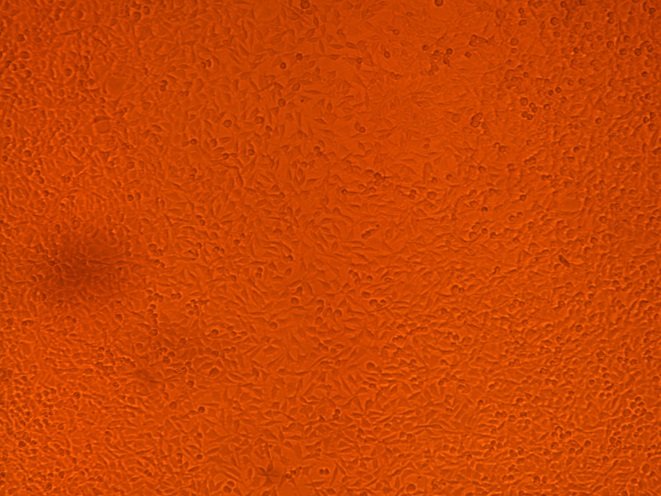

Supplement: Supplementary file 1 — Supplementary Information. [file 41598_2020_68406_MOESM1_ESM.docx]
